# Electric impedance tomography and protective mechanical ventilation in elective robotic-assisted laparoscopy surgery with steep Trendelenburg position: a randomized controlled study

**DOI:** 10.1038/s41598-023-29860-x

**Published:** 2023-02-16

**Authors:** Pasquale Buonanno, Annachiara Marra, Carmine Iacovazzo, Raffaele Merola, Andrea Uriel De Siena, Giuseppe Servillo, Maria Vargas

**Affiliations:** Department of Neurosciences, Reproductive and Odontostomatological Sciences, University of Naples Federico II, Via Pansini 5, 80100 Naples, Italy

**Keywords:** Medical imaging, Prostate, Outcomes research

## Abstract

Electrical impedance tomography (EIT) reconstructs functional lung images and evaluates the variations of impedance during the breathing cycle. The aim of this study was to evaluate the effect of protective mechanical ventilation on ventilation distributions recorded by the EIT during elective robotic-assisted laparoscopy surgery with steep Trendelenburg position. This prospective, randomized single center study included patients with healthy lungs undergoing elective robot-assisted laparoscopic urological surgery in general anesthesia. Patients were randomly assigned to either protective lung ventilation or conventional ventilation. In the protective ventilation group, tidal volume (TV) was set at 6 ml/Kg predicted body weight (PBW), with PEEP 6 cmH_2_O, and recruitment maneuvers (RM) as needed. In the conventional ventilation group, TV was set at 9 ml/Kg PBW, with PEEP 2 cmH_2_O and RM only as needed. Ventilation distribution was assessed using an EIT device. This study included 40 patients in the functional image analysis. Significant differences were found in ventilation distribution in the region of interest (*p* < 0.05). Driving pressure was significantly lower in protective ventilation group (*p* < 0.05). Peak and plateau pressures were not different between the groups while statical significance was found in tidal volume and respiratory rate. EIT may be a valuable tool for monitoring lung function during general anesthesia. During elective robotic-assisted laparoscopy surgery with steep Trendelenburg position, protective mechanical ventilation may have a more homogenous distribution of intraoperative and postoperative ventilation. Larger sample size and long-term evaluation are needed in future studies to assess the benefit of EIT monitoring in operation room.

*Clinical trial registration* ClinicalTrials.gov Identifier: NCT04194177 registered at 11th December 2019.

## Introduction

General anesthesia increases the risk of respiratory complications through the development of atelectasis in dorsal-dependent regions of the lungs, reducing end-expiratory lung volume (EELV) and impairing arterial oxygenation^1,2^.

Postoperative pulmonary complications (PPC) in patients with non-injured lungs are quite common^4^. PPCs represent a heterogeneous group of events such as atelectasis, pulmonary edema, postoperative pneumonia, pleuritis, re-intubation, requirement for postoperative supplemental oxygen^3^. PPCs are associated with increased morbidity and mortality, ICU and hospital length of stay (LOS), and healthcare costs^4–7^. The pathophysiology of PPCs and the preventive strategies to avoid them still require a better understanding among anesthesiologists^8^. Nowadays, it is still unclear if protective lung ventilation with low tidal volumes, positive end-expiratory pressure (PEEP) and recruitment maneuvers may be an effective strategy to reduce the incidence of PPCs in patients under general anesthesia^9–12^. Indeed, during general anesthesia many factors as laparoscopy, pneumoperitoneum, and Trendelenburg position can adversely affect lung function^13^. The reason why is the cranial shift of the diaphragm that promotes the development of atelectasis in dependent lungs regions with a consequent reduction of end-expiratory lung volume (EELV) and oxygenation impairment^13–15^.

Electrical impedance tomography (EIT) is a non-invasive, radiation-free, bedside monitoring system that detects real time regional ventilation changes during perioperative mechanical ventilation; it may be a useful tool to guide individualized protective ventilation strategies to mitigate the adverse effects of anesthesia and surgery on the respiratory system^16^.

With those premises in mind, the aim of this study is to evaluate the effect of protective mechanical ventilation on ventilation distributions recorded by the EIT during elective robotic-assisted laparoscopy surgery with steep Trendelenburg position.

## Materials and methods

This prospective, randomized single center study was approved by the local ethics committee (University of Naples “Federico II”—no132/17) and registered in clinical trial.org where the full protocol was available (NCT04194177—11/12/2019). The study was conducted from March 2020 to April 2022. All procedures were performed in accordance with the Declaration of Helsinki. Informed consent of all patients was obtained before inclusion. Patients were included if they have healthy lungs, 18 years or older, American Society of Anesthesiologists physical status (ASA) ≥ 2, undergoing elective robot-assisted laparoscopic prostatectomy surgery. The presence of chronic pulmonary disease or other obstructive or restrictive disease, congestive heart failure New York Heart Association (NYHA) III/IV, BMI ≥ 35 and ventricular tachyarrhythmias were exclusion criteria.

The random allocation sequence was created by using a web-based encrypted platform with a 1:1 ratio randomization sequence. Patients were assigned to one of the two groups: protective ventilation or conventional ventilation. In both groups, mechanical ventilation was set up at volume-controlled, FiO_2_ was set to 0.4–0.5 to achieve a target oxygen saturation greater than or equal to 92%, and respiratory rate was adjusted to keep End-Tidal carbon dioxide (EtCO_2_) in the normal range of 35–40 mmHg. In the protective ventilation group, tidal volume (TV) was set at 6 ml/Kg predicted body weight (PBW), with PEEP of 6 cmH_2_O, and recruitment maneuvers (RM) as needed. In the conventional ventilation group, TV was set at 9 ml/Kg PBW, with PEEP of 2 cmH_2_O and RM only as needed. At the time of the study there were no standardized guidelines to instruct providers on optimal intraoperative ventilation strategies to reduce the risk PPCs. According to this we decided to set the protective ventilation strategy similarly to the study by Futier et al.^11^, since it was large-scale study mainly performed on the population of abdominal surgical patients that showed better outcomes in protective ventilation group^11^.

General anesthesia was conducted in both groups as follow. After premedication with midazolam (0.05 mg/kg), anaesthesia was induced with sufentanil (0.2 mcg/kg), propofol (2 mg/kg) and rocuronium (0.6 mg/kg) and maintained with desflurane adjusted to achieve minimum alveolar concentration (MAC) 0.8, rocuronium (0.15 mg/kg) train of four (TOF) guided.

**Figure 1:**
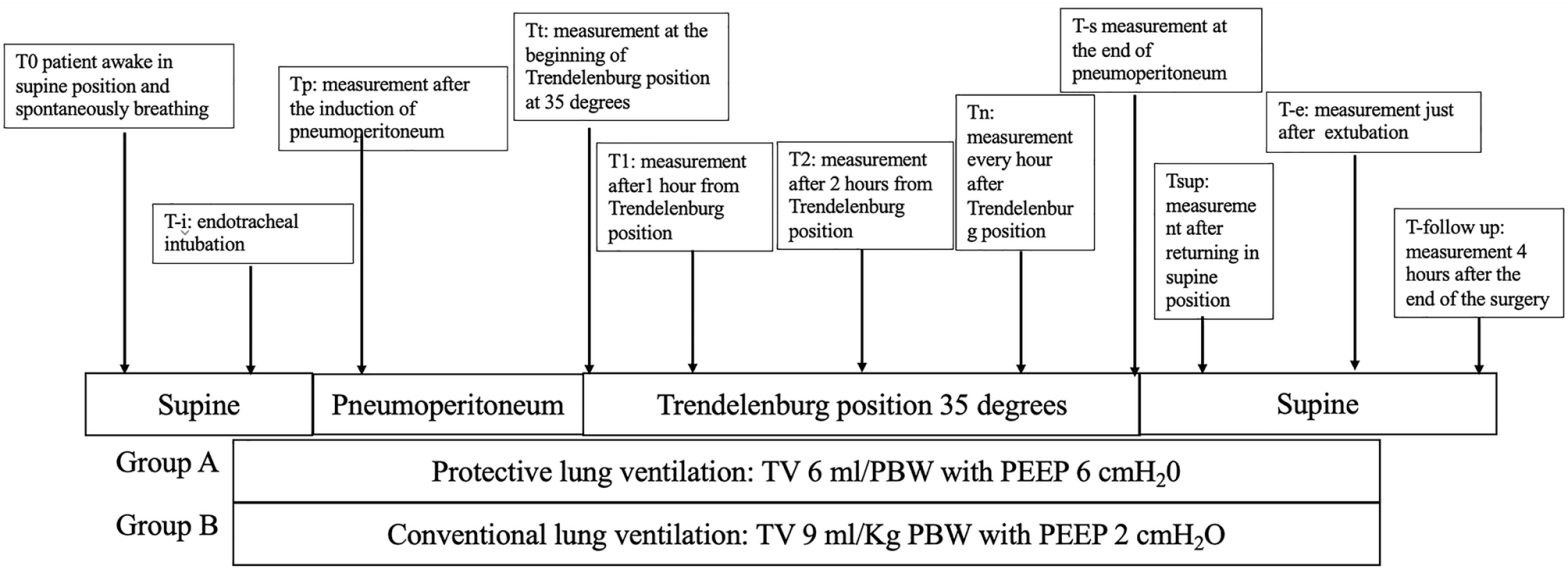
Schematic diagram of study protocol and interventions for both groups.TV: tidal volume, PEEP: end-expiratory positive pressure.

EIT reconstructed functional images with high temporal resolution based on assessing impedance changes during the respiratory cycle^16^. Ventilation distribution was assessed using an EIT device (PulmoVista500; Drager/GoeMFII, Lubeck, Germany) with 16 textile-embedded electrodes placed around the chest along the sixth intercostal space before the beginning of anesthesia (awake patients). We focused our analysis on studying the variation of the amplitude of the ventilation signal in the different lung regions, choosing the tidal variation (amplitude of the global impedance curve) as a parameter. Functional EIT images were analyzed with the Dräger EIT Data Analysis Tool 6.1 (Dräger, Lübeck, Germany) and EITdiag v.1.6 (Dräger, Lübeck, Germany). The Dräger SW EITdiag V1.6 is a dedicated software tool for advanced PC based analysis of EIT data files that have been previously recorded with PulmoVista 500. EITdiag reconstructed EIT images and used various previously published approaches for data interpretation with respect to regional and temporal inhomogeneity of the lung function. The typical workflow that was used for EIT data analysis is the following: EIT data files were loaded; EIT sections of 4 min for analysis were defined; EIT data were reconstructed. For EIT data reconstruction a low-pass filter with a cutoff frequency of 50 min^−1^ was applied to exclude cardiac-related variations. Within the generated tidal images, four horizontal layers for each side were defined as regions of interest (ROIs), and labelled from ventral to dorsal: V (ventral), MV (mid-ventral), MD (mid-dorsal), D (dorsal). For the purpose of this study, we used EIT-diag, a software that compares the amplitude with a reference value. In our case, the chosen reference value was the lung scan “Te” (endotracheal intubation) since, knowing the tidal volume applied by the ventilator, we can presume to obtain the best ventilation signal for that patient. Different perioperative times were chosen to perform the scans on each patient, to be compared with the “Te” reference scan: T0, patient awake in supine position and spontaneously breathing; Te, endotracheal intubation; Tp, after induction of pneumoperitoneum, Tt, at the beginning of Trendelenburg position at 35 degrees; T1, 1 h after Trendelenburg position; T2, 2 h after Trendelenburg position; Tn, any additional hour after Trendelenburg position; Ts, at the end of pneumoperitoneum; Tsup, returning in supine position; Te, extubation; T-follow up, 4 h after the end of the surgery (Fig. 1). Peak pressure (Ppeak), plateau pressure (Pplat), driving pressure (DP), end-tidal CO_2_ (etCO_2_), respiratory rate (RR) and tidal volume (TV) were recorded during the surgery. The primary outcome was the distribution of ventilation in the region of interest between the considered groups. The secondary outcome were the evaluations of DP, Ppeak, Pplat, etCO_2_, RR and TV recorded during the surgery between the considered groups.

### Ethical approval

Ethical committee of university of Naples Federico II approved the study protocol (123/17). Written informed consent was obtained from each patient or next of kin.

## Statistical analysis and sample size

Sample size was calculated according to the primary outcome of the study by Shono et al.^17^. To reach a power of 80% with an alpha error 0.05, we had to include 12 patients for each group. Data were analyzed by Shapiro test to investigate the normal distribution; parametric data were presented as mean and standard deviation, non-parametric data as median and interquartile range. ANOVA was used for continuous variables, and proportions were compared with χ^2^ or Fisher exact test, as appropriate. *p* values < 0.05 were considered statistically significant. Analyses were performed with SPSS 20.0 (SPSS Corp, Chicago, IL).

## Results

**Figure 2:**
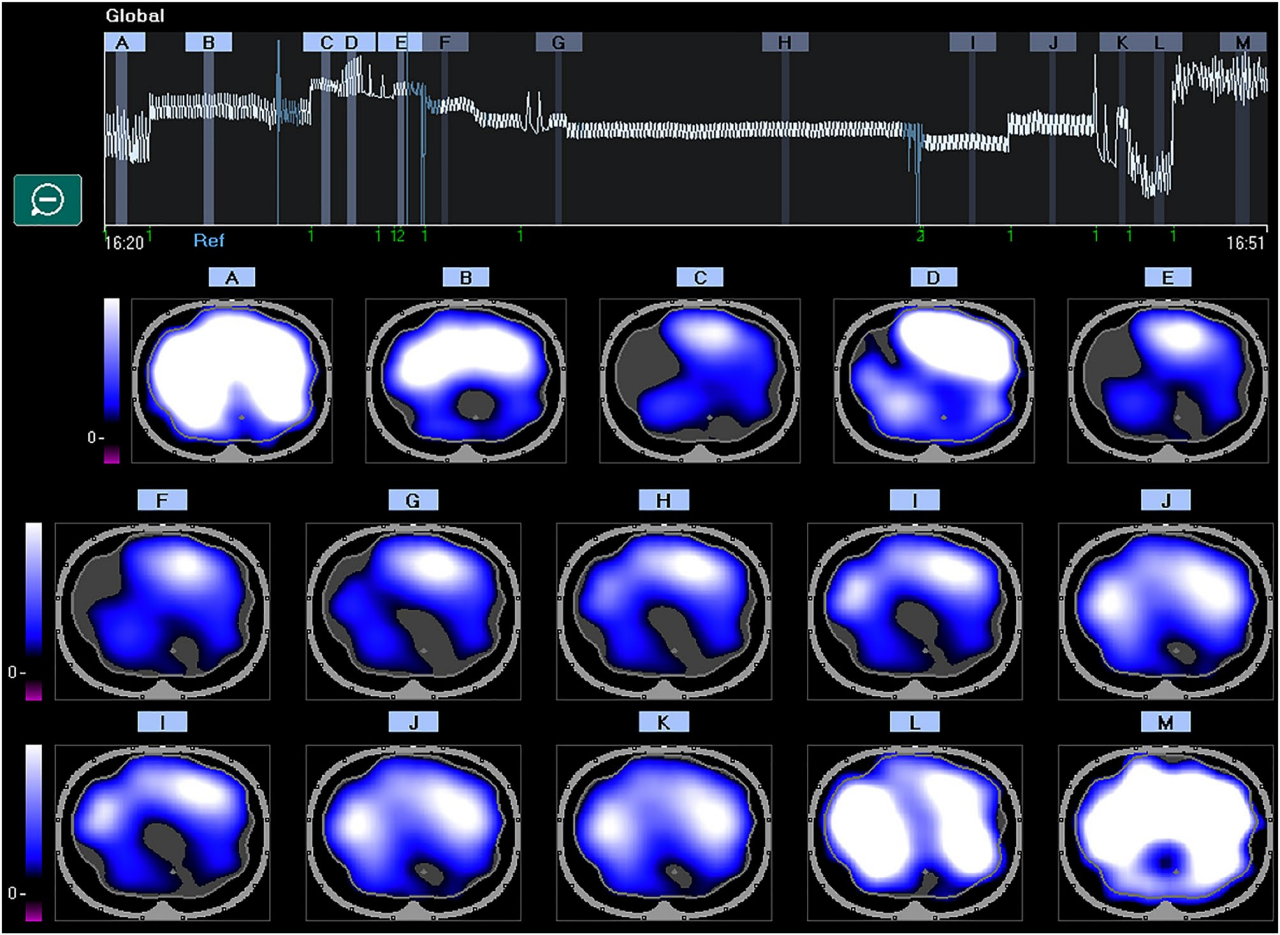
EIT distribution of lung ventilation during protective mechanical ventilation. Regional distribution of tidal breath is visualized with a color scale based on calculated impedance changes during one breath. Brighter color (corresponding to large impedance change) shows a well-ventilated area. Darker color (small impedance change) shows a less ventilated area.

**Figure 3:**
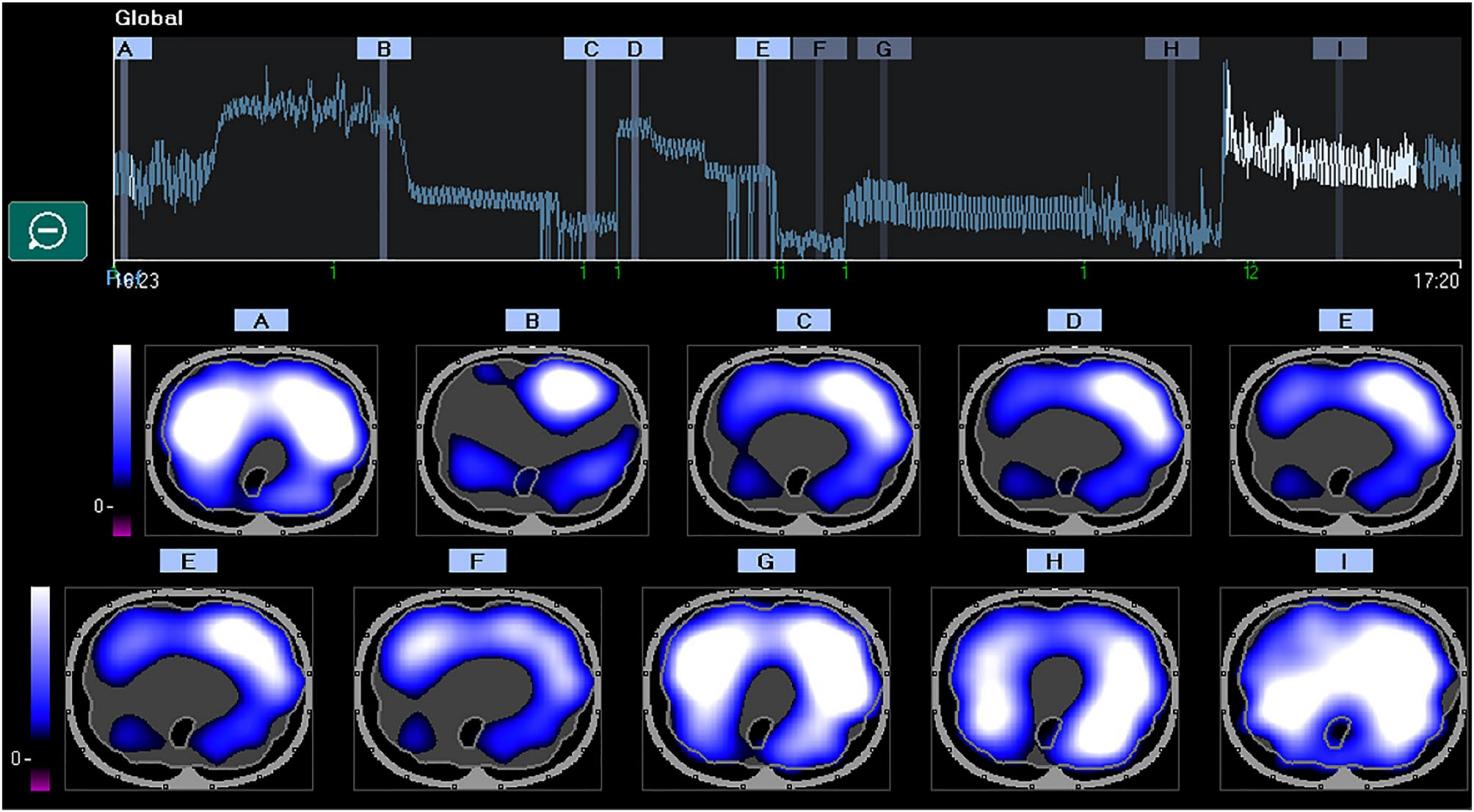
EIT distribution of lung ventilation during conventional mechanical ventilation. Regional distribution of tidal breath is visualized with a color scale based on calculated impedance changes during one breath. Brighter color (corresponding to large impedance change) shows a well-ventilated area. Darker color (small impedance change) shows a less ventilated area.

**Figure 4:**
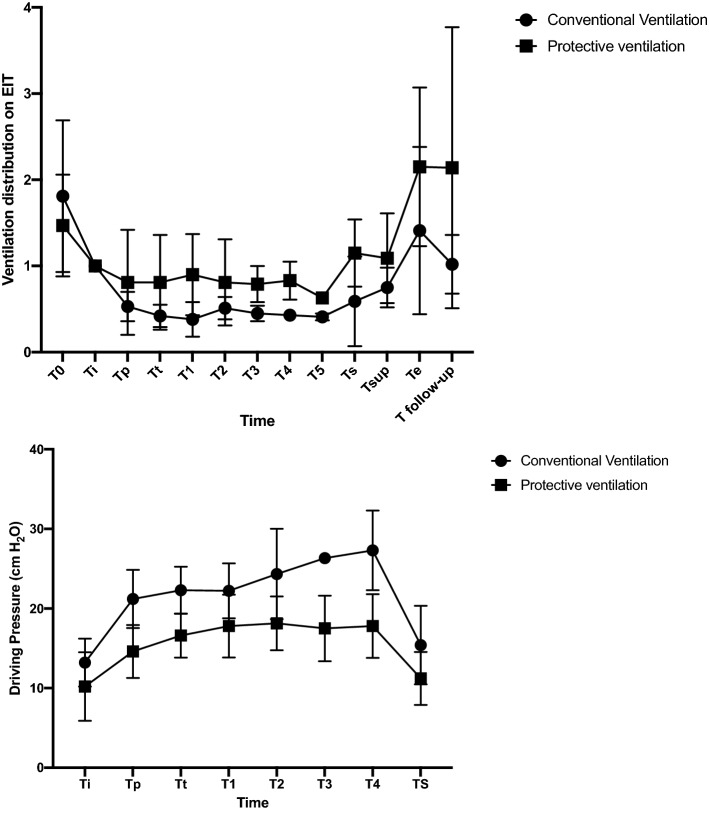
(**A**) EIT distribution of lung ventilation during protective and conventional mechanical ventilation over time. (**B**) Distribution of driving pressure ventilation during protective and conventional mechanical ventilation over time.

Twenty-one patients were included in each group (Fig. 1). Table 1 showed the characteristics of included patients. No significant difference existed between the baseline characteristics of two groups. One patient’s EIT data per group was found unanalyzable due to technical problems such as low signal quality due to the electrocautery interference. Thus, we included 40 patients in the functional image analysis. Significant differences were found in ventilation distribution in the region of interest. The protective ventilation group had a homogenous ventilation distribution from Tt to Tsup (*p* < 0.05). Interestingly the protective ventilation group had a statistically significant ventilation distribution even at Te and T-follow up (*p* = 0.04 and *p* = 0.04, respectively). Figures 2 and 3 showed representative images of EIT in both groups. During surgery the ventilation was homogeneously distributed in the protective ventilation group than in conventional groups, even if a drop in ventilation distribution was seen in both groups after the induction of anesthesia and pneumoperitoneum (Figs. 2 and 3). Figure 4 showed the changes in ventilation distribution within the region of interest and in driving pressure in both groups. Driving pressure was significantly lower in protective ventilation group from Tt to Ts (*p* < 0.05) (Fig. 4).

**Table 1:** Patient demographic and clinical features.

	Protective ventilation (n = 20)	Conventional ventilation (n = 20)	*p* value
Age (years)	54 ± 9	51 ± 8	
Height (cm)	173.9 ± 7.74	174.1 ± 6.98	0.95
Weight (kg)	79.7 ± 9.63	75.9 ± 8.79	0.369
BMI (kg/m^2^)	27.8 ± 3.6	25.4 ± 4.5	0.45
O_2_ Saturation (%)	99 ± 0.82	99.44 ± 1.67	0.463
Anesthesia time (minute)	269 ± 43	273 ± 65	0.864
Surgery time (minute)	238 ± 31	252 ± 66	0.560
ASA (n)			
I	0	0	0.331
II	8	9	
III	12	11	
IV	0	0	
V	0	0	
Respiratory infection within 30 days of surgery (n)	0	0	0.99
Smokers (n)	3	3	0.966

Data are number of patients (n) or mean standard deviation (SD).

*ASA* American Society of Anesthesiologists, *cm* centimeters, *kg* kilograms.

Ppeak and Pplat pressures were not different between the groups while statical significance was found in tidal volume and respiratory rate and lung compliance at certain time (Table 2).

**Table 2:** Respiratory parameters recorded during the study.

	End tidal CO_2_		Respiratory rate (breaths/min)		Tidal volume (ml)		FiO_2_ (%)		Peak Pressure (cmH_2_O)		Plateau pressure (cmH_2_O)		Lung compliance (ml/cmH_2_O)	
	Prot	Conv	Prot	Conv	Prot	Conv	Prot	Conv	Prot	Conv	Prot	Conv	Prot	Conv
At the endotracheal intubation	35 ± 3	33 ± 3	12 ± 1*	12 ± 1	452 ± 75*	648 ± 111	46 ± 3	46 ± 3	17 ± 4	17 ± 4	16 ± 4	15 ± 3	30.1 ± 3	43.9 ± 10*
After induction of pneumoperitoneum	35 ± 3	33 ± 3	14 ± 1*	13 ± 3	483 ± 64*	640 ± 120	47 ± 4	46 ± 3	22 ± 2	24 ± 4	21 ± 3	23 ± 4	34.8 ± 9.9	30.8 ± 7.2
At the beginning of Trendelenburg position at 35°	38 ± 1	33 ± 3*	15 ± 2*	13 ± 3	476 ± 59*	633 ± 123	47 ± 4	47 ± 3	24 ± 2	25 ± 4	23 ± 3	24 ± 3	34.4 ± 9.9	30.4 ± 7
1 h after Trendelenburg position	36 ± 2	34 ± 2*	15 ± 2*	12 ± 2	484 ± 67*	631 ± 89	47 ± 4	46 ± 3	25 ± 3	25 ± 4	24 ± 4	24 ± 3	28.5 ± 7.9	28.9 ± 5.9
2 h after Trendelenburg position	37 ± 2	33 ± 2*	15 ± 2*	11 ± 2	480 ± 83*	691 ± 107	47 ± 4	47 ± 3	25 ± 4	26 ± 5	25 ± 4	22 ± 6	26.7 ± 5	36.7 ± 9.9*
3 h after Trendelenburg position	38 ± 1	33 ± 1*	16 ± 4*	10 ± 2	508 ± 85*	724 ± 101	48 ± 3	48 ± 3	25 ± 5	28 ± 1	25 ± 4	28 ± 1	29.7 ± 5.2	27.5 ± 4.3
4 h after Trendelenburg position	34 ± 0	34 ± 1	14 ± 0*	9 ± 1	600 ± 53	764 ± 90	47 ± 4	50 ± 0	24 ± 1	29 ± 3	24 ± 2	27 ± 2	37.5 ± 2.4	29.4 ± 3.4
End of pneumoperitoneum	36 ± 2	32 ± 2*	16 ± 3*	11 ± 2	487 ± 69*	652 ± 99	47 ± 4	47 ± 3	19 ± 2	19 ± 5	17 ± 3	17 ± 5	47.2 ± 20	45.2 ± 12.8

*Prot* protective, *conv* conventional, *ml* milliliters.

± : SD. *p < 0.05.

## Discussion

In this randomized controlled study we found that lung protective ventilation, applied during elective robotic-assisted laparoscopy surgery with steep Trendelenburg position, improved ventilation distribution recorded by EIT and reduced driving pressure.

To our knowledge, this is the first randomized controlled study evaluating the use of EIT to monitor protective ventilation during robotic-assisted laparoscopy surgery with a steep Trendelenburg position. Several studies suggested that pneumoperitoneum and Trendelenburg position can adversely affect lung function and ventilation^13–15^. Particularly, robot-assisted surgery with pneumoperitoneum and steep Trendelenburg position increased intra-abdominal and airway pressure and induced a cephalad displacement of the diaphragm, which result in decreased functional residual capacity and lung compliance, ventilation-perfusion mismatch and atelectasis^13–15^. In this situation the use of lung protective ventilation with a low tidal volume, low PEEP and recruitment maneuvers have shown to minimize these consequences while the benefit of high PEEP has not yet established^17^. EIT may help anesthesiologists to evaluate lung function during general anesthesia by imaging breath by breath changes in ventilation distribution and to tailor mechanical ventilation on patient needs^16^. Previous studies in patients undergoing laparoscopic cholecystectomy with monitoring of ventilation distributions by EIT demonstrated that intraoperative and postoperative tidal volume distributions were more homogenous in patients with a PEEP 10 cmH_2_O than in the group with 0 cmH_2_O^18–20^. Otherwise, during robotic gynecological surgery, high PEEP (8 cmH_2_0) did not contribute to a significant increase in dorsal portions of the regional ventilation distribution, evaluated by EIT, when compared with the low PEEP (4 cm H_2_O)^21^. In an observational study on patients undergoing robotic assisted radical prostatectomy, EIT was able to identify and quantify circumscribed areas, like silent spaces within healthy, lungs that received little or no ventilation during general anesthesia, pneumoperitoneum, and different body positions^16^.

Our study sustained the evidence that protective mechanical ventilation with an adequate PEEP level improved the distribution of ventilation evaluated by the EIT during general anesthesia. Even if a drop in tidal volume distribution was seen in both groups after induction and anesthesia, the protective ventilation group had a homogeneous distribution of ventilation from the beginning of the Trendelenburg position to come back in supine position. Therefore, the protective ventilation group showed a better ventilation distribution also at the extubation and follow-up. Indeed, only after the extubation we found that EIT-derived parameters and driving pressure came back to the pre-anesthesia levels suggesting that persistent atelectasis and a ventilation–perfusion mismatch were present until the end of general anesthesia.

We did not use zero PEEP as a control group to minimize the negative effects of anesthesia and pneumoperitoneum and Trendelenburg position on lung function^22^. Our established PEEP level was well tolerated since the driving pressure in the lung protective ventilation group was significantly lower than in the conventional ventilation group^22,23^.

This study had several strengths. First, this is the first randomized controlled study evaluating the use of EIT during elective robotic-assisted laparoscopy surgery with steep Trendelenburg position. Second, the recruitment of 40 patients exceeds the estimated sample size (24 patients), thus the study is adequately powered. Third, this study may be a proof of concept that EIT may be used as respiratory monitoring in operation room.

This study had also several limitations. First, the electrical interferences caused by the use of the electrocautery affected EIT measurements and impaired their interpretation, making it necessary to exclude these breaths. Second, EIT belt positioning was a key factor in measurement consistency, since its position was not standardized. Third, the possible clinical impact of our intervention was not evaluated since was not an aim of this study protocol.

## Conclusions

EIT may be a valuable tool for monitoring lung function during general anesthesia. During elective robotic-assisted laparoscopy surgery with steep Trendelenburg position, protective mechanical ventilation may have a more homogenous distribution of intraoperative and postoperative ventilation. Larger sample size and long-term evaluations are needed in future studies to assess the benefit of EIT monitoring in operation room.

## Data Availability

Data and materials are available from the corresponding author after request.
